# Assessment of ESGO Quality Indicators in Cervical Cancer Surgery: A Real-World Study in a High-Volume Chinese Hospital

**DOI:** 10.3389/fonc.2022.802433

**Published:** 2022-01-25

**Authors:** Yan Ding, Xuyin Zhang, Junjun Qiu, Jianfeng Zhang, Keqin Hua

**Affiliations:** ^1^ Department of Gynecology, Obstetrics and Gynecology Hospital of Fudan University, Shanghai, China; ^2^ Shanghai Gynecology Quality Control Center, Shanghai, China

**Keywords:** cervical cancer, oncological outcome, quality assurance, quality of treatment, gynecologic oncologists

## Abstract

The ESGO developed a list of fifteen quality indicators for cervical cancer surgery in order to audit and improve clinical practice in 2020. However, data from the developing countries with high incidence rates of cervical cancer is still lacking. Therefore, we conducted a retrospective study of 7081 cases diagnosed as cervical cancer between 2014 and 2019 in a Chinese single center according to the quality indicators proposed by ESGO. A total of 5952 patients underwent radical procedures, with an average of 992.0 per year. All surgeries were performed or supervised by a certified gynecologic oncologist as surgical qualification grading system has been established. Compared with the low-volume group, patients in the high-volume group (≥15 cases/year) had a shorter hospital stay (*P*<0.001), more free surgical margins (*P*=0.031), and less complications (*P*<0.001), but the 5-year recurrence-free survival and overall survival rates were similar (*P*>0.05). Treatment was not planned at a multidisciplinary team meeting but with the consultation system. The required preoperative workup was incomplete in 19.7% of patients with pelvic MRI and 45.7% of patients with PET-CT. A total of 1459 (20.6%) patients experienced at least one complication after surgery. The CDC grade IIIb or higher complications occurred in 80 patients, accounting for 5.5% complications. The urological fistula rate within 30 postoperative days were 0.3%. After primary surgical treatment, 97.4% patients had clear vaginal and parametrial margins. After restaging FIGO 2009 to FIGO 2018 system, 14.7% patients with a stage T1b disease were T-upstaged. After a median follow-up of 42 months, recurrence occurred in 448 patients, and 82.1% patients recurred within 2 years. The 2-year RFS rate of patients with pT1b1N0 was 97.3% in 2009 FIGO staging system. Lymph node staging was performed in 99.0% patients with a stage T1 disease. After a primary surgical treatment for a stage pT1b1N0 disease, 28.3% patients received adjuvant chemoradiotherapy. Above all, most of quality indicators reached the targets, except four quality indicators. The quality indicators of ESGO should be popularized and applied in China to guarantee quality of surgery.

## Introduction

Cervical cancer is the fourth most common cancer in women worldwide, with an estimated 604,000 new cases and 342,000 deaths in 2020 (1). In China, the age-standardized incidence and mortality rates of cervical cancer have been constantly increasing over last 20 years, with 109,741 new cases and 59,060 deaths of cervical cancer in 2020, approximately accounting for 18% and 17% that of the world respectively (2). Surgery is the preferred treatment for patients with early-stage cervical cancer. Clear evidence was found that implementation of a quality improvement program helped to reduce both morbidity and costs, and improve the quality of life of cancer patients. Moreover, the quality of surgical care has been shown to improve outcomes in patients with other malignances such as breast cancer, lung cancer, gastric cancer, colorectal cancer, soft tissue sarcoma, ovarian cancer, and so on (3–7). Thus, it is likely that implementation of a quality management program could improve survival of patients with cervical cancer. In 2020, the European Society for Gynecologic Oncology (ESGO) then developed a list of fifteen quality indicators (QIs) in an easy and practicable way in order to audit and improve the surgical treatment of cervical cancer (8).

To our knowledge, few studies assessed the quality of cervical cancer surgery based on the ESGO list of quality indicators. A retrospective study including 1156 cases from 126 institutions belonging to 29 European countries evaluated the ESGO quality indicators for surgical treatment of cervical cancer (9). And another multicenter retrospective study in Europe assessed the oncological outcomes of 239 patients diagnosed with cervical cancer according to the quality indicators (10). However, data from the developing countries with high incidence rates of cervical cancer is still lacking. Therefore, we conducted a retrospective real-world study involving patients diagnosed as cervical cancer between 2014 and 2019 in the Obstetrics and Gynecology Hospital of Fudan University, Shanghai, China, so as to audit the surgery quality of cervical cancer in this high-volume single center according to the quality indicators proposed by ESGO.

## Material and Methods

### Study Population

It was a retrospective study under real-world conditions. The study was approved by the Institutional Review Board (IRB) of the Obstetrics and Gynecology Hospital of Fudan University (No.2021-15). All patients who diagnosed with cervical cancer and underwent surgical treatment from January 1, 2014 to December 31, 2019 in the Obstetrics and Gynecology Hospital of Fudan University were included. Exclusion criteria were: ① no surgical management during the period of inclusion, ② just biopsy or conization for diagnose but not for surgical treatment, and ③ undergoing other surgical treatment but not related to the cervical cancer therapy.

### Data Collection

Using the International Classification of Diseases Tenth Revision (ICD-10) code C53.9 or the diagnosis of “cervical cancer” as the keyword for the search, data were extracted from the hospital information system and the outpatient information system. The tumors were classified according to the Federation International of Gynecology and Obstetrics (FIGO) staging system. Between 2014 and 2018, patients were diagnosed with the 2009 FIGO staging system, while the 2018 FIGO staging system began to be used in 2019 (11, 12). In principle, patients underwent operations based on different stages according to the National Comprehensive Cancer Network (NCCN) guidelines at that time. All the procedures were accomplished with the use of a uterine manipulator and without vaginal closure and tumor exclusion before the colpotomy before 2018. But after the report of the Laparoscopic Approach to Cervical Cancer (LACC) trial, the uterine manipulator was banned, and the tumor was enclosed before the colpotomy in the hospital. Some of the patients with bulky (≥4 cm) stage IB or IIA cervical carcinoma were treated by neoadjuvant chemotherapy at the discretion of the treating gynecologist. The patients received paclitaxel and platinum for 1-2 courses, and then underwent surgical treatment. We extracted the information of complications through the identical information of patients and reanalyzed them according to the Clavien-Dindo classification (CDC) system (13) and the comprehensive complication index (CCI) (14). The CCI values were computed from the CCI calculator at website (http://www.assessurgery.com).

A patient was considered to be treated by a certified gynecologist if her gynecologist had a corresponding surgery qualification. The surgical qualification grading system has been established in the hospital since 2013 according to the provisions of the National Health Administration, which is similar to the Endoscopic Surgical Skill Qualification System (ESSQS) in Japan (15). According to the system, surgical qualifications are classed into four grades and authorized by the Surgical Qualification Examination Committee. Surgical Grade IV are subdivided into pelvic lymphadenectomy (IVa), radical hysterectomy (IVb), and paraaortic lymphadenectomy (IVc). Since the minimum required number of radical procedures per year was 15, we classified those who qualified for surgical Grade IVb and performed more than 15 cases of radical procedures per year as the high-volume surgeons, while those who qualified for surgical Grade IVb but performed <15 cases/year radical procedures, or those who did not qualify for surgical Grade IVb and performed radical procedures under supervision as the low-volume surgeons.

After surgery, patients underwent adjuvant therapy if they presented any high-risk factors (positive margin, parametrial involvement, or lymph node metastasis) or intermediate-risk factors met the Sedlis criteria (16) or the “four-factor model” (17). According to the NCCN guidelines, patients were followed up every 3 months for 2 years, every 6 months for the next 3 years, and once per year thereafter. The follow-up information was recorded in the follow-up information system and can be obtained after searching for the identical information of the patient. The last follow-up date was December 2020. Recurrence-free survival (RFS) was defined as the length of time (in months) from the primary surgery to initial diagnosis of recurrence or date of last follow-up. Overall survival (OS) was calculated (in months) as the difference between the primary surgery date and the date of death from cervical cancer or last contact, whichever came first.

### Statistical Analyses

Statistical analyses were performed with SPSS v23.0 (IBM Corp., Armonk, NY). Student’s t-test or analysis of variance (ANOVA) was used to compare continuous variables, whereas chi-square test was used to compare categorical variable. Oncological outcomes, RFS and OS were calculated with the Kaplan-Meier method, with differences in the probability of survival analyzed with the log-rank test. Differences were considered to be statistically significant at *P <*0.05.

## Results

A total of 7081 patients with diagnosis of cervical cancer between January 2014 and December 2019 were finally enrolled as the study population. The clinical characteristics of all patients were shown in **Table 1**. The mean age of all patients was 48.1 years old. Majority of patients (99.0%) were FIGO stage <IIB, and more than half patients (51.0%) were stage IB1. A total of 6891 (97.3%) surgeries were performed by minimally invasive surgery. Of these, 6489 (94.2%) patients had a laparoscopic approach, and 402 (5.8%) patients had robotic surgery. Only 135 (1.9%) patients underwent by laparotomy. Another 55 (0.8%) patients underwent transvaginal repeat cone biopsy because of fertility sparing. The surgical procedure was described as radical surgery in 5952 (84.0%) cases. A total of 5985 (84.5%) patients underwent lymphadenectomy, mostly (89.8%) with pelvic lymphadenectomy. While only 24 (0.4%) cases underwent sentinel lymph node biopsy.

**Table 1:** Clinical characteristic of patients with cervical cancer in different years.

Variables	N = 7081
Age (years), median (range)	48.1 ± 10.0 (8-84)
FIGO 2009 stage, n (%)	
IA1	1203 (17.0)
IA2	182 (2.6)
IB1	3614 (51.0)
IB2	640 (9.0)
IIA1	884 (12.5)
IIA2	485 (6.9)
≥IIB	73 (1.0)
Surgical approach, n (%)	
Laparoscopy	6489 (91.6)
Robotic surgery	402 (5.7)
Laparotomy	135 (1.9)
Transvaginal surgery	55 (0.8)
Type of surgical resection, n (%)	
Radical surgery	5952 (84.0)
Radical hysterectomy	5653 (95.0)
Modified radical hysterectomy	188 (3.2)
Trachelectomy	73 (1.2)
Parametrectomy	38 (0.6)
Cone biopsy	55 (0.8)
Hysterectomy	1068 (15.1)
Local recurrence resection	6 (0.1)
Type of lymph node dissection, n (%)	5985 (84.5)
Sentinel lymph node biopsy	24 (0.4)
pelvic lymphadenectomy	5373 (89.8)
pelvic and para-aortic lymphadenectomy	588 (9.8)

All results of the ESGO quality indicators in the hospital year by year were shown in **Table 2**.

**Table 2:** Evaluation of the ESGO quality indicators in the hospital.

	Quality indicators	Target	Total result	2014	2015	2016	2017	2018	2019	*P-value*
1	Radical procedures performed per year	≥30	992.0 ± 207.3	705	841	915	1101	1126	1264	<0.001
2	Certified surgical specialist	100%	100%	100%	100%	100%	100%	100%	100%	
3	Ongoing clinical trials	≥1	3.3 ± 2.7	1	1	2	3	5	8	0.002
4	Multi-disciplinary team meeting	100%	0%	0%	0%	0%	0%	0%	0%	
5	Required pre-operative investigation	100%	54.3%	40.1%	43.2%	50.8%	52.2%	61.0%	78.5%	<0.001
6	Required elements in surgical reports	100%	100%	100%	100%	100%	100%	100%	100%	
7	Required elements in pathology reports	≥90%	100%	100%	100%	100%	100%	100%	100%	
8	Structured prospective reporting of the follow-up and 30-day postoperative morbidity	≥90%	90%	90%	90%	90%	90%	90%	90%	
9	Urological fistula rate within 30 days after a radical parametrectomy	≤3%	0.3% (19/5952)	0.3%	0.4%	0.4%	0.2%	0.3%	0.4%	0.919
10	Negative vaginal and parametrial margins	≥97%	97.4% (6897/7081)	97.8%	97.8%	97.5%	97.4%	97.6%	96.6%	0.314
11	T-upstaged after surgery in T1b disease	<10%	14.7% (626/4254)	12.4%	14.8%	12.6%	17.9%	16.5%	13.0%	0.010
12	Recurrence rate at 2 years in patients with pT1b1N0	<10%	2.7%	2.7%	4.2%	3.6%	3.7%	1.7%	0.6%	0.002
13	Lymph node staging in T1 disease	≥98%	99.0% (3467/3501)	99.8%	98.9%	99.3%	99.5%	99.4%	97.7%	0.001
14	Counseling about fertility-sparing treatment	100%	100%	100%	100%	100%	100%	100%	100%	
15	Adjuvant chemoradiotherapy in pT1b1N0 disease	<15%	28.3% (876/3098)	24.1%	24.5%	23.4%	30.8%	32.2%	31.0%	0.001

### Quality Indicators Related to Caseload in the Center, and Training and Experience of the Surgeon

QI 1 is a structural indicator, which means the number of radical procedures in cervical cancer performed per center per year. The optimal target is ≥30 cases and the minimum required target is ≥15 cases. As shown in **Table 2**, a total of 5952 patients underwent radical procedures, with an average of 992.0 ± 207.3, which significantly exceeded the optimal target. The number of radical procedures increased significantly year by year (*P*<0.001).

QI 2 is a process indicator, which means surgery performed or supervised by a certified gynecologic oncologist or a trained surgeon dedicated to gynecological cancer (accounting for 80% of his or her practice) or having completed an ESGO-accredited fellowship. The target is 100%. This indicator was performed 100% in our center.

**Figure 1:**
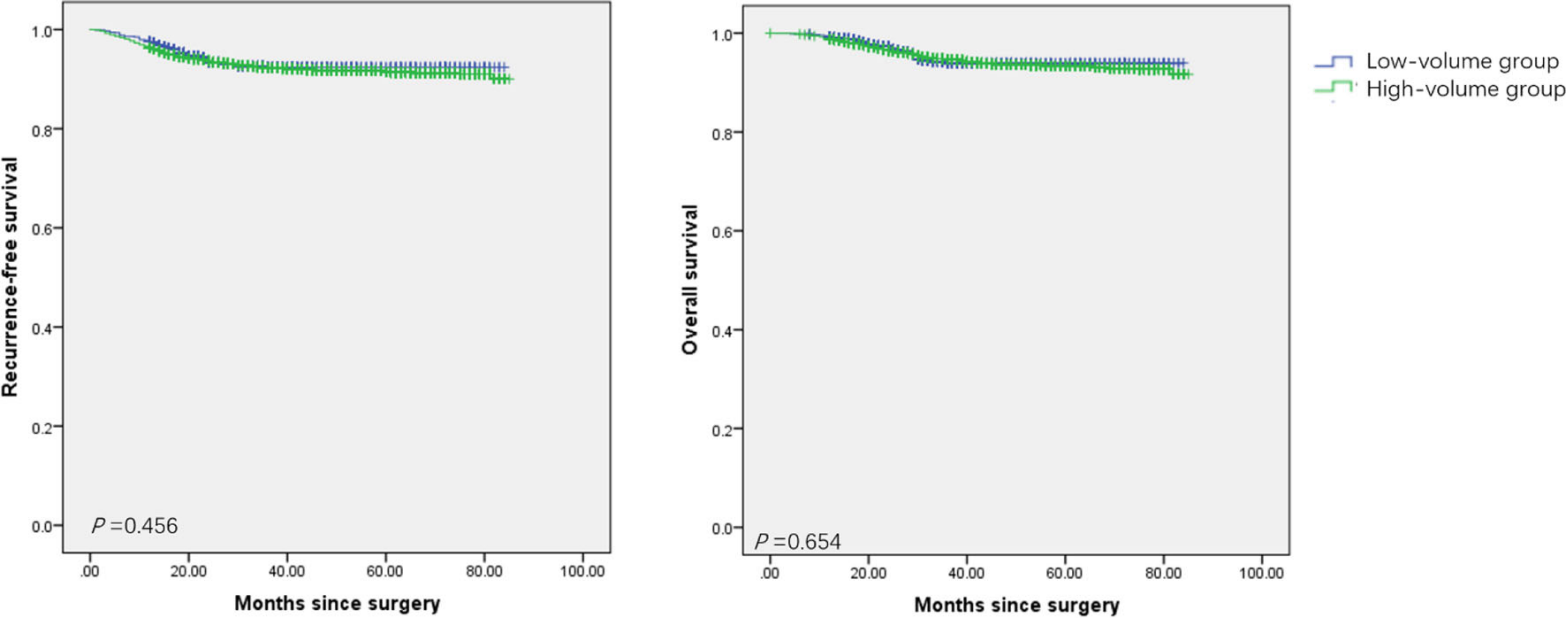
Survival of patients with cervical cancer treated with radical procedures. **(A)** the Kaplan-Meier estimates of recurrence-free survival between the high-volume group and the low-volume group. **(B)** the Kaplan-Meier estimates of overall survival between the high-volume group and the low-volume group.

A total of 40 surgeons underwent radical procedures, while 36 of these qualified for surgical Grade IVb. Among them, 18 surgeons were divided into the high-volume group as they underwent radical procedures ≥15 cases/year, with a total of 5016 (84.3%) patients. Ten surgeons who qualified for the robotic radical hysterectomy were all in the high-volume group, and underwent 390 cases since 2015. As seen in **Table 3**, patients in the high-volume group were younger (48.3 *vs* 49.7, *P*<0.001), and more likely to be stage IB1 or ≥IIB (*P*<0.001). They had a higher incidence of superficial stromal infiltration (41.9% *vs* 38.6%, *P*=0.042), no lymphovascular space incision (LVSI) (55.5% *vs* 51.5%, *P*=0.023), and free surgical margins (93.1% *vs* 91.2%, *P*=0.031). Furthermore, the patients in the high-volume group had a shorter hospital stay (11.0 *vs* 12.5 days, *P*<0.001), and less intraoperative complications as well as postoperative severe complications (*P*<0.001), especially in the incidence of urological injury and fistula. But there was no significant difference between the two groups in the cumulative 5-year RFS rates (91.4% *vs* 92.4%, *P*=0.456) and OS rates (93.3% *vs* 91.4%, *P*=0.654) (**Figure 1**).

**Table 3:** Comparison of clinical, pathologic and operative characteristics between the high-volume and the low-volume groups.

	Total (n = 5952)	High-volume (n = 5016)	Low-volume (n = 936)	*P-value*
Age (years)	48.5 ± 10.0	48.3 ± 9.9	49.7 ± 10.2	<0.001
FIGO 2009 stage (n,%)				<0.001
IA1	127 (2.1)	111(2.2)	16 (1.7)	
IA2	174 (2.9)	141 (2.8)	33 (3.5)	
IB1	3588 (60.3)	3076 (61.3)	512 (54.7)	
IB2	636 (10.7)	537 (10.7)	99 (10.6)	
IIA1	884 (14.9)	731 (14.6)	153 (16.3)	
IIA2	479 (8.0)	360 (7.2)	119 (12.7)	
≥IIB	64 (1.1)	60 (1.2)	4 (0.5)	
Type of surgery (n,%)				<0.001
Laparoscopy	5440 (91.4)	4541 (90.5)	899 (96.0)	
Robotic surgery	390 (6.6)	390 (7.8)	0 (0.0)	
Laparotomy	122 (2.0)	85 (1.7)	37 (4.0)	
Histological type (n,%)				0.660
SCC	4753 (79.9)	3996 (79.7)	757 (80.9)	
AC	715 (12.0)	614 (12.2)	101 (10.8)	
ASC	376 (6.3)	315 (6.3)	61 (6.5)	
Other type	108 (1.8)	91 (1.8)	17 (1.8)	
Tumor size, mm (n,%)				0.059
≤20	2418 (40.6)	2069 (41.2)	349 (37.3)	
(20-40]	2237 (37.6)	1873 (37.3)	364 (38.9)	
>40	1297 (21.8)	1074 (21.4)	223 (23.8)	
Stromal infiltration (n,%)				0.042
<1/3	2464 (41.4)	2103 (41.9)	361 (38.6)	
[1/3 -2/3)	287 (4.8)	230 (4.6)	57 (6.1)	
≥2/3	3201 (53.8)	2683 (53.5)	518 (55.3)	
LVSI (n,%)				0.023
No	3267 (54.9)	2785 (55.5)	482 (51.5)	
Yes	2685 (45.1)	2231 (44.5)	454 (48.5)	
Parametrial involvement (n,%)				0.850
No	5522 (92.8)	4655 (92.8)	867 (92.6)	
Yes	430 (7.2)	361 (7.2)	69 (7.4)	
Uterine involvement (n,%)				0.956
No	4951 (83.2)	4173 (83.2)	778 (83.1)	
Yes	1001 (16.8)	843 (16.8)	158 (16.9)	
Vaginal involvement (n,%)				0.163
No	4071 (68.4)	3449 (68.8)	622 (66.5)	
Yes	1881 (31.6)	1567 (31.2)	314 (33.5)	
Ovarian involvement (n,%)				0.353
No	5952 (99.5)	4995(99.6)	930 (99.4)	
Yes	27 (0.5)	21 (0.4)	6 (0.6)	
Lymph node metastasis (n,%)				0.519
No	4664 (78.4)	3938 (78.5)	726 (77.6)	
Yes	1288 (21.6)	1078 (21.5)	210 (22.4)	
Number of lymph node (n)	22.1 ± 7.7	22.0 ± 7.7	22.3 ± 7.9	0.201
Surgical margin status (n,%)				0.031
Free margins	5524 (92.8)	4670 (93.1)	854 (91.2)	
Free but close margins (<5mm)	50 (0.8)	40 (0.8)	10 (1.1)	
Positive margins (pre-invasive disease)	203 (3.4)	172 (3.4)	31 (3.3)	
Positive margins (invasive disease)	175 (2.9)	134 (2.7)	41 (4.4)	
NACT				0.910
No	5809 (97.6)	4895 (97.6)	914 (97.6)	
Yes	143 (2.4)	121 (2.4)	22 (2.4)	
Adjuvant treatment (n,%)				0.185
No	2655 (44.6)	2256 (45.0)	399 (42.6)	
Yes	3297 (55.4)	2760 (55.0)	537 (57.4)	
Operative time (min)	172.6 ± 65.6	171.2 ± 63.6	176.4 ± 70.7	0.214
Estimated blood loss (ml)	233.3 ± 192.1	232.7 ± 187.8	236.4 ± 213.7	0.594
Hospital stays (day)	12.3 ± 5.8	11.0 ± 5.6	12.5 ± 5.8	<0.001
Intraoperative complications (n,%)	31 (0.5)	11 (0.2)	20 (2.1)	<0.001
Ureteral injury	8 (0.1)	0 (0.0)	8 (0.9)	
Bladder injury	13 (0.2)	7 (0.1)	6 (0.6)	
Bowel injury	6 (0.1)	2 (0.0)	4 (0.4)	
Vascular injury	3 (0.1)	2 (0.0)	1 (0.1)	
Obsturator nerve injury	1 (0.0)	0 (0.0)	1 (0.1)	
Postoperative complications (n,%)	29 (0.5)	12 (0.2)	17 (1.8)	<0.001
Bowel obstruction	2 (0.0)	0 (0.0)	2 (0.2)	
Hemorrhage	3 (0.1)	2 (0.0)	1 (0.1)	
Vesicovaginal fistula	5 (0.1)	2 (0.0)	3 (0.3)	
Ureteral fistula	14 (0.2)	6 (0.1)	8 (0.9)	
rectovaginal fistula	1 (0.0)	0 (0.0)	1 (0.1)	
Deep venous thrombosis	4 (0.1)	2 (0.0)	2 (0.2)	

LVSI, lymphovascular space incision; SCC, squamous cell carcinoma; AC, adenocarcinoma; ASC, adenosquamous carcinoma; NACT, neoadjuvant chemotherapy.

### Quality Indicators Related to the Overall Management

QI 3 is a structural indicator, which means the center participating in ongoing clinical trials in gynecological cancer. The target is ≥1. Twenty clinical trials had been conducted from 2014 to 2019, with an average of 3 clinical trials ongoing every year. The target was performed 100%.

QI 4 is a process indicator, which means treatment discussed at a multi-disciplinary team (MDT) meeting. The target is 100%. But there was no MDT meeting in our hospital before 2020. Instead, the consultation system was performed. The target was totally not performed.

QI 5 is a process indicator, which means required preoperative investigation. The target is 100%. As seen in **Table 4**, all patients underwent pelvic examination, and the average of clinical tumor size was 20.0 mm. All patients underwent pelvic ultrasound, with an average size of 20.6 mm. But pelvic MRI with contrast was performed in 80.3% of patients with stage ≥ IB1, and the mean tumor diameter measured by MRI was 24.2mm. Whole-body PET-CT or chest/abdomen/pelvic CT was performed in 54.3% of patients in locally advanced cervical cancer and higher. Actually, the main problem of the preoperative workup was the whole-body imaging. Fortunately, the completion rate of imaging was increasing year by year (*P*<0.001). All patients in locally advanced cervical cancer and higher performed urinary examination. Nearly all patients underwent a cervical biopsy except 7 (0.1%) patients were found incidentally after hysterectomy. As indicated, 2466 (99.7%) patients underwent cone biopsy except nine patients who were so elder with cervical atrophy that difficult to operate.

**Table 4:** Characteristics of the preoperative assessment of patients.

Items	Number (%)
Pelvic examination	7081
Yes	7081 (100.0)
No	0 (0.0)
Tumor clinical size, mm	20.0 ± 17.7
≤20	3521 (49.7)
>20	3560 (50.2)
Preoperative pathology	7081
Cervical biopsy	7075 (99.9)
No cervical biopsy	6 (0.1)
Cervical conization as indicated	2473
Yes	2466 (99.7)
No	7 (0.3)
Pelvic ultrasound	7081
Yes	7081 (100.0)
No	0 (0.0)
Max diameter of US, mm	20.6 ± 19.7
Pelvic MRI with *contrast* in FIGO stage ≥ IB1	5696
Yes	4575 (80.3)
No	1121 (19.7)
Max diameter of MRI in FIGO stage ≥ IB1, mm	24.2 ± 19.1
Whole-body PET-CT or chest/abdomen/pelvic CT in locally advanced cervical cancer and higher	2082
Yes	1130 (54.3)
No	952 (45.7)
Urinary ultrasound or CTU in locally advanced cervical cancer and higher	2082
Yes	2082 (100.0)
No	0 (0.0)

MRI, magnetic resonance imaging; CT, computed tomography; PET-CT, positron emission tomography/computed tomography; CTU, computed tomography urography.

### Quality Indicators Related to Recording Pertinent Information

QI 6 is a process indicator, which means minimum required elements in surgical reports. The target is 100%. All required elements as defined in the ESGO-ESTRO-ESP guidelines were present in the patient surgical report. The target was performed 100%.

QI 7 is a process indicator, which means minimum required elements in pathology reports. The target is ≥90%. Three tumor dimensions were all measured, with the average maximum tumor size of 23.8 ± 19.3 mm. All the other required elements as defined in the ESGO-ESTRO-ESP guidelines were present in the patient pathology report, as seen in the **Table 3**. The target was performed 100%.

QI 8 is an outcome indicator, which means structured prospective reporting of the follow-up and 30-day post-operative morbidity using a validated surgical complication scoring system. The optimal target is ≥90% and the minimum required target is that selected cases are discussed at morbidity and mortality conferences. The target was performed 90% in our hospital. A total of 1459 (20.6%) patients experienced at least one complication after surgery. The type, occurrence time, reason, and management of complications as well as recovery of the patient were all reported. Every complication which leaded to organ injury or function permanent damage and even death of a patient would be discussed and defined as grade of medical events in the meeting. However, the CDC system or the CCI had never been used in the hospital. Therefore, the data in the complication reporting system were reviewed and reanalyzed in **Table 5**. Bladder injury (0.2%) was the most common intraoperative complications. Leg lymphedema (17.5%), bladder dysfunction (9.8%), and fever (7.2%) were the most common postoperative complications. The CDC grade IIIb or higher complications occurred in 80 (1.1%) patients, accounting for 5.5% complications. The mean CCI was 18.2 ± 8.0.

**Table 5:** Complications analysis according to the CDC and the CCI.

CDC grade	Number of CDC	CCI scores	Number of CCI
Grade I	2281	8.7	231
		12.2	375
		15.0	154
		17.3	122
Grade II	580	20.9	69
		22.6	115
		24.2	98
		29.6	136
		30.8	45
		32.0	34
Grade IIIa	25	26.2	9
		27.6	11
		33.5	5
Grade IIIb	53	33.7	48
		39.7	5
Grade IVa	2	51.7	1
		58.1	1
Grade IVb	0		
Grade V	0		

CDC, the Clavien–Dindo classification; CCI, the comprehensive complication index.

### Quality Indicators Related to the Quality of Surgical Procedures

QI 9 is an outcome indicator, which means urological fistula rate within 30-post-opetative days after a radical parametrectomy in the preceding 3 years. The target is ≤3%. As seen in **Table 3**, a total of 40 (0.7%) patients had urologic complications in 6 years. Furthermore, urinary injury and bladder injury occurred in 0.4% (22/5952) and 0.3% (18/5952) of patients, respectively. Of these, 19 patients (0.3%) had urological fistula after radical procedures. The incidence of urological fistula was similar every year.

QI 10 is an outcome indicator, which means proportion of patients after primary surgical treatment who have clear vaginal and parametrial margins in the preceding 3 years. The target is ≥97%. In the center, 6897 (97.4%) cases had clear surgical margins after primary surgical treatment in 6 years. There was no significant difference every year.

QI 11 is an outcome indicator, which means proportion of patients with a stage T1b disease T-upstaged after surgery. The target is <10%. All patients were reclassified following the 2018 FIGO staging system based on pathology report. As seen in **Table 6**, a total of 2527 (35.7%) patients were restaged. Of these, 2107 patients were upstaged: 1300 (61.7%) due to lymph node metastasis, 453 (21.5%) due to vaginal involvement, 232 (11.0%) due to tumor size, 117 (5.6%) due to parametrial involvement and 5 (0.2%) due to ovarian involvement or distant metastasis. Of these, 14.7% (626/4254) patients with a stage T1b disease were T-upstaged after surgery, which did not reach the target.

**Table 6:** Shift in stage for cervical cancer patients from FIGO 2009 to FIGO 2018.

2009 FIGO	2018 FIGO												
	IA1	IA2	IB1	IB2	IB3	IIA1	IIA2	IIB	IIIC1	IIIC2	IVA	IVB	Total
IA1	1154	17	23	1		3			5				1203
IA2		169	10	1		2							182
IB1			1749	855	120	280	62	31	493	23	1		3614
IB2			20	84	174	42	64	26	215	15			640
IIA1			80	100	21	321	60	30	263	9			884
IIA2			7	22	40	36	107	30	215	26		2	485
IIB				2	1	5	6	11	29	7	1	1	63
IVA											3		3
IVB												7	7
Total	1154	186	1889	1065	356	689	299	128	1220	80	5	10	7081

**Figure 2:**
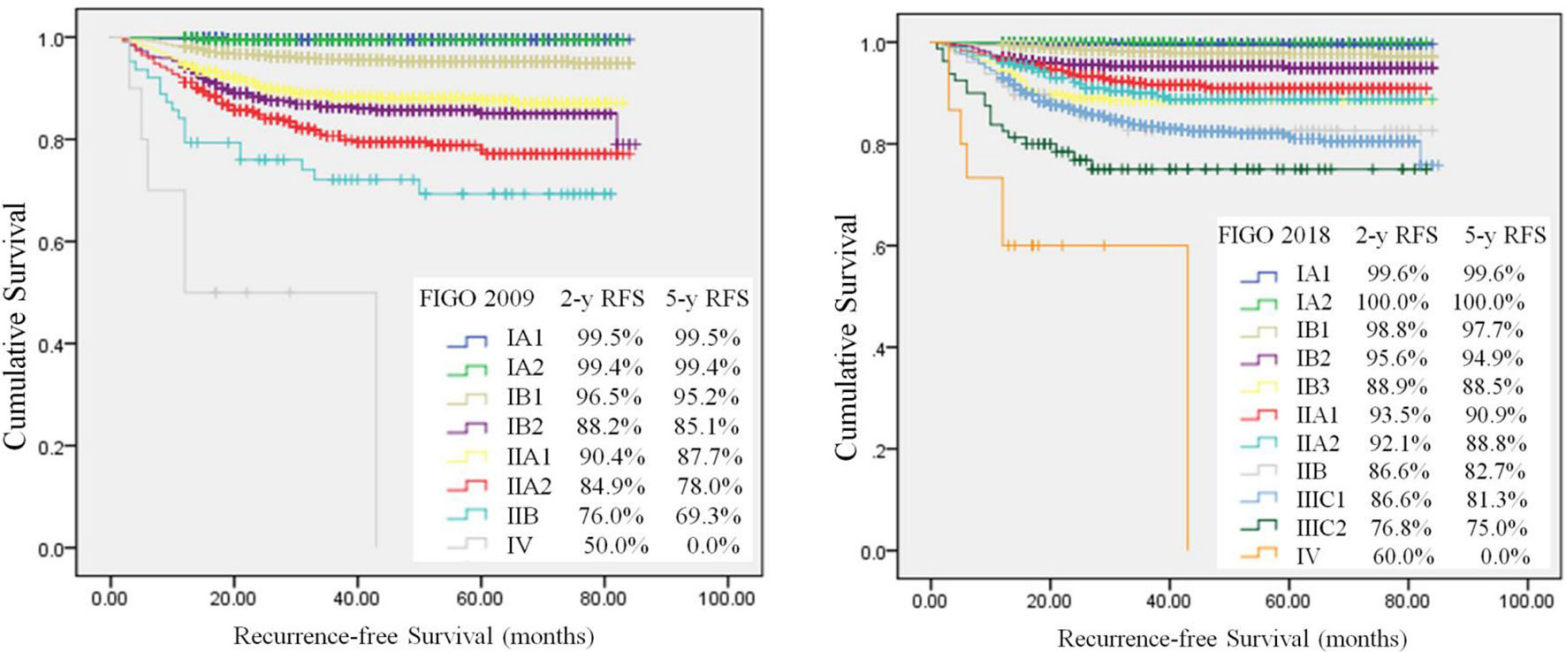
Kaplan-Meier analysis of recurrence–free survival. Differences between use of 2009 FIGO (log-rank test, *P*<0.001) and 2018 FIGO (log-rank test, *P* < 0.001) staging systems.

QI 12 is an outcome indicator, which means recurrence rate at 2 years in patients with a stage pT1b1 with negative lymph nodes after primary surgical treatment. The target is <10%. After a median follow-up of 42 months (range 0-85), 5844 (82.5%) patients remained free of disease, 448 (6.3%) patients occurred recurrence, and 316 (4.5%) patients had died. The 5-year RFS and OS rate were respectively 91.9% and 94.3%. The 2-year RFS and OS rate were respectively 93.4% and 95.0%. Most of patients (82.1%) recurred within 2 years after surgery. The RFS rate was analyzed in the 2009 and 2018 FIGO staging systems by Kaplan-Meier analysis (**Figure 2**). The 2-year RFS of patients with T1b1N0 was 97.3%, and the 5-year RFS rate was 96.2% in the 2009 FIGO staging system. While in the 2018 FIGO staging system, the 2-year RFS of patients with stage IB1and IB2 was 97.6%, and the 5-year RFS rate was 96.7%. The recurrence rate was significantly reduced after 2018 (*P*=0.002). Compared minimally invasive radical hysterectomy to open surgery for early-stage cervical cancer, there was no significant difference in patients with T1b1N0 in the 2-year RFS rate (97.3% *vs* 96.7%, *P*=0.721), or the 5-year RFS rate (96.2% *vs* 96.7%, *P*=0.721). Similarly, there was no significant difference in patients with T1 disease in the 2-year RFS rate (95.4% *vs* 95.1%, *P*=0.613), or the 5-year RFS rate (94.0% *vs* 91.0%, *P*=0.613).

### Quality Indicators Related to the Compliance of Management With the Standards of Care

QI 13 is an outcome indicator, which means proportion of patients with a stage T1 disease treated by primary surgery who have undergone lymph node staging according to the ESGO-ESTRO-ESP guidelines. The target is ≥98%. Before surgery, all patients with stage T1 were scheduled to undergo lymph node staging according to guidelines. During surgery, five patients were found to upgrade from stage IA1 to stage ≥IA2 according to the results of frozen sections, yet the agents of the patients refused to expand the operative extent but to choose radiation. After surgery, the final pathologic diagnosis showed that 25 patients were upstaged from high grade squamous intraepithelial lesions, carcinoma in situ, or stage IA1 without LVSI. Furthermore, four patients were found cervical cancer unexpectedly according to the postoperative pathology. Therefore, a total of 34 patients did not undergo lymph node staging. In other words, there were 99.0% (3467/3501) patients with T1 disease underwent lymph node staging. The patients in 2019 had the lowest rate of lymph node staging (*P*=0.001).

QI 14 is a structural indicator, which means counseling about a possibility of fertility-sparing treatment (FST). The target is 100%. All eligible patients with stage T1 were counseled about the possibility of FST. A total of 128 patients underwent FST.

QI 15 is a structural indicator, which means proportion of patients receiving adjuvant chemoradiotherapy after a primary surgical treatment for a stage pT1b1pN0 disease. The target is <15%. There were 3098 patients with pT1b1N0 according to the 2009 FIGO staging system. Of these, 1100 (35.5%) patients with high risk or intermediate risk required adjuvant therapy. In fact, 876 out of 1100 (28.3%) patients received adjuvant therapy at last. There was no significant difference between the completed group and the uncompleted group in 5-year RFS rates (96.3% *vs* 93.3%, *P*=0.097) as well as in OS rates (94.9% *vs* 91.5%, *P*=0.077). Whereas 897 out of 2954 (30.4%) patients with stage IB1 and IB2 according to FIGO 2018 staging system required adjuvant chemoradiotherapy, while 685 (23.2%) patients received adjuvant therapy actually. The rate of patients with pT1b1N0 receiving adjuvant therapy varied significantly from year to year (*P*=0.001), but neither reached the target.

## Discussion

Implementation of a quality management program in surgery has a major impact on survival of cancer patients. The ESGO developed a list of quality indicators for cervical cancer surgery with the aim of auditing clinical practice in 2020. Therefore, we retrospectively analyzed the quality of cervical cancer surgery for 7081 cases from 2014 to 2019 in our hospital according to the ESGO quality indicators for self-assessment and improvement. It showed that most of quality indicators achieved the target, except four quality indicators which were MDT, preoperative investigation, T-upstaged and adjuvant therapy. To the best of our knowledge, this is the first study comprehensively evaluating the quality of surgical treatment of cervical cancer in a single institution, especially in such a high-risk area of cervical cancer as China. Moreover, the large sample size and relatively long duration of follow-up are also the strength of research.

### The Quality of Hospital Management

As the incidence rate of cervical cancer has been increasing in China, nearly 1000 patients of cervical cancer every year were treated in the hospital, which contributed to almost the largest number in Shanghai. The effect of hospital volume on outcomes of surgery is related to a surgeon’s skill and experience as well as the supporting team (8). Radical surgery performed by a gynecologic oncologist is recommended to be the preferred treatment modality in early-stage disease by ESGO. Different from the sub-specialty training program in gynecologic oncology in Europe, the surgical qualification grading system has been established for years in China. Increasing studies showed that high surgical volume of cervical cancer was a favorable prognostic factor for operative outcomes and peri-operative complication rates (18–21). Latest studies stressed that a steady trend of reduction in disease recurrence risk is associated with increased surgeon experience (22, 23). The 3-year RFS was significantly lower at the beginning of a surgeon’s learning path compared to the time he had been adequate experience. Hence, we classified the surgeons who were at the beginning of learning path or did not perform the radical treatment frequently into the low-volume group. We found that there was no significant difference in treatment outcomes no matter what surgeons were in the learning path. This may because surgeons in either group could meet adequate surgical standards after training of the surgical qualifications grading system. However, surgeons who had adequate experience conferred significant benefit in terms of a shorter hospital stay, more free surgical margins, and lower risks of complications.

### The Quality of Management Before Surgery

An accurate diagnosis guides patient management and informs prognosis. In our study, not all the patients reached the goals, especially whole-body assessment in patients of locally advanced cervical cancer. There may be some reasons. First, surgeons may not be fully aware of the importance of imaging. Second, imaging diagnoses were not accurate interpretation so that surgeons could not get effective information. Third, the examination of MRI, CT or PET-CT was expensive for some of patients in China. Adequate clinical staging with imaging and vaginal assessment is crucial for decisions on choice of treatment and tailoring of surgery. On contrary, inaccurate preoperative assessment led to increasing rates of postoperative upgrading and adjuvant chemoradiotherapy. Fortunately, the completion rate of imaging was increasing year by year. Overall preoperative evaluation should be more emphasized and improved in our hospital.

Multi-disciplinary care is internationally recognized as best practice in treatment planning and care. However, the consultation system but not MDT was performed in our hospital if the patient was in sophisticated or dangerous situation and need to discuss with different departments. For example, all patients who met the standard of FST were informed and discussed with anesthesiologists, obstetricians, or endocrinologists to provide a whole-process treatment plan. The MDT system should be established in our hospital.

### The Quality of Management During Surgery

In the study, only 1.0% patients did not perform lymph node staging in the primary surgery due to upstaging or incidental finding of cervical cancer. Accurate preoperative evaluation could avoid missing lymph node staging. Identification of sentinel lymph nodes and its ultra-staging is highly recommended because it increases staging accuracy (24–26). But sentinel lymph nodes biopsy was attempted just in 24 patients. The ABRAX trial recently showed that if lymph node involvement is detected intra-operatively, further pelvic lymph node dissection and radical hysterectomy should be avoided (27). But all the patients who were found positive lymph nodes underwent radical hysterectomy further in the hospital, and even the patients with ≥IIB underwent radical hysterectomy. There may be some reasons. First, most Chinese patients had a deeply rooted prejudice that surgery was the best treatment for cancer. Second, compared with radiologist, gynecologists were more likely to recommend surgery. Third, the problem of side effects of radiotherapy, especially the long-term side effects, has not been solved, which directly affects the subsequent quality of life, especially for young patients. Fourth, it had been a great challenge for doctors to treat the recurrence after radiotherapy. Fifth, lack of radiotherapy equipment leads to the choice of surgery for patients but not wait for radiotherapy.

### The Quality of Management After Surgery

The surgical complications had been still reported in the ranking system in the hospital, while the ESGO recommend the CDC system and CCI, which are widely applied in many fields of surgery, including cervical cancer (13, 14, 28, 29). Thereafter, the CDC and the CCI should be introduced in the hospital so as to improve patient management. Urologic complication is an important quality indicator because it may lead to increased rates of reoperation and readmission, an increased length of stay, and increased litigations. The incidence of urologic complications varies from 0% to 6.0% (ureteral), 0.1% to 3.0% (bladder) and 0.4% to 4.5% (fistula) (30). In our hospital, urologic complications were seen in 0.7% of the cohort, and the postoperative genitourinary fistulas was 0.3%. The significant lower incidence rate may be attributed to the patient characteristics, and the surgeon’s operative experience. Previous studies showed that the proportion of urinary fistulas was twice that of the intraoperative urinary injuries (30, 31). However, we found that the proportion of intraoperative and postoperative of urinary injuries was similar. This may be due to the prophylactic placement of ureteral stent during operation and the control of postoperative infection, which reducing the ischemic damage of ureter and bladder. Furthermore, the incidence of ureteral injury and bladder injury was also similar in the study, which was consistent with previous studies (32, 33).

The study showed that most of patients recurred within 2 years after surgery, and the 2-year recurrence rate of patients with pT1b1N0 was 2.7% in our study, which was similar to previous studies that the recurrence rate of patients with pT1b1N0 was less than 10% within 2 years of primary surgery, irrespective of the neo-adjuvant or adjuvant treatment strategy (8, 34, 35). Furthermore, the LACC trial in 2018 (34) showed that minimally invasive radical hysterectomy was associated with lower RFS rates than open radical hysterectomy (91.2% *vs* 97.1%, HR 3.74). A resent respective study (35) also found that the recurrence rate in the open surgery was significantly lower than that in minimally invasive radical hysterectomy (7.5% *vs* 9.1%, *P*=0.43). However, in this study there was no significant difference in the RFS rate for patients with T1b1N0 or T1 between open surgery and minimally invasive radical hysterectomy. This may be related to the small number of patients who underwent open surgery. In fact, patients had been carefully and fully counseled about the surgical outcomes and oncologic risks of the different surgical approaches after the LACC trial according to the NCCN guidelines. But open surgery was still limit (1.9%). Here are some reasons. Minimally invasive surgery was associated with reduced blood loss, shorter hospital stays, and fewer postoperative complications compared to open surgery. Some measures such as no use of uterine manipulator and tumor enclosing before colpotomy had been taken to improve tumor-free technology in the minimally invasive radical hysterectomy. A multicenter, retrospective, observational cohort study showed that avoiding the uterine manipulator and using maneuvers to avoid tumor spread at the time of colpotomy in minimally invasive surgery was associated with similar outcomes to open surgery (36). Several prospective clinical trials on the outcome of different surgical approaches have been also launched in the hospital. It turned out that the recurrence rate was significantly lower after 2018. A pilot study of forty-eight patients with early-stage who underwent vaginal-assisted gasless laparoendoscopic single-site radical hysterectomy also showed no relapsed in the hospital (37). Prospective studies and longer follow-up periods should be performed to further evaluate the oncological outcomes.

In the study, more than 30% patients with T1b1N0 were required adjuvant therapy. On the contrast, 20% patients chose to observe rather than receiving adjuvant therapy. In fact, observation is an alternative option in experienced teams when adequate type of radical hysterectomy has been performed according to the ESGO-ESTRO-ESP guidelines (38). Actually, there was no significant difference between the completed group and the uncompleted group in 5-year RFS rates in the study. So accurate preoperative assessment, appropriate treatment options, adequate radical surgery, and close follow-up will reduce the incidence of adjuvant therapy.

### Limits of the Study

There are several limitations of this study. First, it is a retrospective study and there may be unrecognized bias. Second, the objectivity of the current study is dependent on accurate charting and documentation, which could be incomplete or inaccurate sometimes. Third, some patients received adjuvant chemotherapy or radiotherapy after surgery. But not all patients received adjuvant therapy in the same institution, so the effect of variation in irradiation technique and chemotherapeutic regimens cannot be eliminated. Fourth, our data only reflect a single center experience. Further investigation at multiple centers is needed.

## Conclusion and Future Research

In this Chinese cohort, we found that most of quality indicators achieved the goals even before the publication of the ESGO quality indicators, except four quality indicators which concentrated on MDT, preoperative investigation, T-upstaged and adjuvant therapy after operation. In future, the MDT, the CDC system, and the CCI should be established. Overall preoperative evaluation should be emphasized and improved in the hospital. Multicenter prospective studies and longer follow-up periods should be performed to further evaluate the oncological outcomes. Furthermore, such a study could conveniently be conducted at a hospital level in order to draw up an inventory of strategies and recommend lines of improvement. The ESGO quality indicators should be popularized and applied in China to guarantee quality of surgery and homogeneous treatment throughout the country to patients with cervical cancer.

## Data Availability Statement

The original contributions presented in the study are included in the article/supplementary material. Further inquiries can be directed to the corresponding authors.

## Ethics Statement

The studies involving human participants were reviewed and approved by the institutional review board of the Obstetrics and Gynecology Hospital, Fudan University. Written informed consent was exempted because of a retrospective study.

## Author Contributions

KH and JZ conceived and designed the study. YD designed the study, performed the statistical analysis, and drafted the manuscript. JZ, XZ, and JQ performed the data collection and analysis. All authors read and critically revised the manuscript for intellectual content and approved the final manuscript.

## Funding

This research received financial support from Shanghai Municipal Health Commission (2021WB01).

## Conflict of Interest

The authors declare that the research was conducted in the absence of any commercial or financial relationships that could be construed as a potential conflict of interest.

The reviewer, XJ, has declared a shared parent affiliation with the authors at the time of review.

## Publisher’s Note

All claims expressed in this article are solely those of the authors and do not necessarily represent those of their affiliated organizations, or those of the publisher, the editors and the reviewers. Any product that may be evaluated in this article, or claim that may be made by its manufacturer, is not guaranteed or endorsed by the publisher.
